# Report on the Diseases of Cincinnati in the Spring of 1828

**Published:** 1828-07

**Authors:** 


### II. ORIGINAL.

12. *Report on the Diseases of Cincinnati in the Spring of* 1828, *concluded from page* 176—*June number.*—4. *Ague and Fever.*—Cases of Ague occurred this Spring, as usual, and, indeed, had presented themselves though, less frequently throughout the Winter. In almost every instance the individual affected, had experienced some form of autumnal fever in the preceding year, or two years before. We feel justified in the conclusion, therefore, that our vernal intermittents are in reality but relapses. Their predisposing cause, at least, is the morbid impress of the foregoing year. For their immediate reproduction an exciting cause, in general, some violation of the *non-naturals* (to revive the quaint nomenclature of our professional ancestors) was necessary.

The symptoms which they exhibited were various. In some cases they assumed the character of periodical headache or *sun pain;* in others the paroxysm consisted chiefly in pains and achings of the muscles and periosteum of the long bones; in some there was but little chilliness and a great deal of yawning; in others the cold stage amounted to a shake; in all, the hot stage was either slight, or, if violent, of short duration. The type was, in different cases, both tertian and quotidian. In some instances the complaint assumed a congestive or malignant aspect, but it was in aspect only, for we met with no case in which it proved fatal, as the malignant intermittents of Autumn are wont to do. A few individuals had repeated attacks; the distance between them, and the number of persons affected becoming less and less, as the weather became warmer. The subjects of it were chiefly emigrants from aguish situations, or inhabitatns of Cincinnati who had travelled the preceding year.

Vernal intermittents are often complicated with enlargements of the liver and spleen, especially the latter; which are the consequences of repeated attacks of fever; and by augmenting the debility of the patient, increase his liability to relapses.

In the treatment of the vernal intermittents of the present, as of preceding springs, we have not found a debilitating method efficacious. We met with no case, in which *bloodletting* was required. *Emetico-cathartics* were, in general, requisite; if, indeed, a deranged state of the secretory functions of the liver and gastro-enteritic membrane call for that class of medicines. We were accustomed to administer them; but are by no means satisfied that they are always necessary. Our reliance was on *tonics* and *stimulants;* and, in the results of their liberal exhibition, we were seldom disappointed. Our practice was to commence their administration at the earliest possible period of the attack; and to continue it through the paroxysm as well as the intermission. We have, in some cases, employed the *bark*, in substance, united with powdered *cloves*, and the *bitartrate of potash.* In this composition the spice reconciles the stomach to the impress of the bark, while the hydragogue and diuretic virtues of the bitartrate, contribute, signally, to the prevention, or removal of the visceral enlargements of which we have spoken.

And here we may take occasion to say, that for the cure of the enlarged spleen or ague cake, we know of no more successful method, than the administration of tonics and diuretics, combined. Of the former the bark is the most powerful and appropriate: of the latter, we have found the nitrate, and bitartrate of potash and the squill, most successful. In these cases, general corroboration and increased secretion, are the conditions requisite to a cure; and, if it be attempted by either method, alone, the success will be less perfect. Whatever plan is adopted, however, we must persevere, for weeks, and, sometimes, even for months, before a cure will be effected. But increased renal secretion is not the only kind that will answer in these cases. Much may be done by exciting the liver; for which purpose, the *pil. hydrargyri* may be administered in suitable portions, at night, the bark being given by day. Or, we may act on the intestinal mucus membrane, with jalap or the compound powder of that root, united, with the cinchona. But we must return to the subject of Ague and Fever.

Within the last three months we have repeatedly substituted the *sulhpate of quina*, for the bark itself, in vernal intermittents, and have found it successful. In several instances, we have united it with the *sulphate of morphia*. The following formula has answered, in most cases:

℞j. sulph. quin. gr. viij.
—— morph. gr. j.
Mix and divide into 8 powders.

One to be taken every two or three hours, without any regard to the paroxysm. In some instances the proportion of sulph. morphiæ was found too large; and in others the sulph.

quinæ too small. Mitigation of fever, tranquility, and perspiration, generally followed on the use of this compound. In a few cases, we saw it ejected, apparently in consequence of the gastric irritation which itself excited; but, by perseverance, the stomach, at length, became quiet, and the desired effect was produced.

We shall only add, that we have found an attention to the state of the skin, and the daily use of small quantities of bark, or some other bitter, the most effectual means of preventing relapses.

13. *Diarrhœa.*—In common years, we are not presented with cases of diarrhœa, till near summer. In the present spring it was otherwise. Early in May, when there was no intense heat, nor any obvious atmospheric cause; no inconsiderable number of persons, chiefly young men, were attacked with this malady. It was not in general attended with vomiting. Some of them had considerable fever, and in some, the symptoms strongly inclined to those of dysentery. We heard of no fatal case. It continued to prevail for two or three weeks. The treatment required was simple. An emetico-cathartic, followed by the compound powder of ipecac, when there was fever, or by a pill of opium, when no fever was present, generally moderated all the symptoms, and sometimes even effected a cure. In other cases it was necessary to administer a second cathartic after the suspension of the peristaltic action by the opiate, and then to quiet the system by the action of a second anodyne. After which, recovery in general took place, without further difficulty.

In the latter part of May, cholera and dysentery began to show themselves, as usual; but being strictly summer epidemics, we shall omit any notice of them, until we come to make report of the diseases of that season.

